# A Novel Rabies Vaccine Based on a Recombinant Bovine Herpes Virus Type 1 Expressing Rabies Virus Glycoprotein

**DOI:** 10.3389/fmicb.2022.931043

**Published:** 2022-06-08

**Authors:** Caiquan Zhao, Jie Gao, Yongzhi Wang, Lina Ji, Hui Qin, Wei Hu, Yang Yang

**Affiliations:** ^1^The State Key Laboratory of Reproductive Regulation and Breeding of Grassland Livestock, School of Life Sciences, Inner Mongolia University, Hohhot, China; ^2^School of Biological Science and Technology, Baotou Teachers' College, Baotou, China

**Keywords:** rabies virus, bovine herpesvirus type I, vaccine, glycoprotein, dendritic cell, virus-neutralizing antibody

## Abstract

Rabies is a highly prevalent zoonotic disease and a public health threat worldwide. Currently licensed rabies vaccines are effective but less is known which would protect cattle. This study describes the construction of a novel recombinant bovine herpes virus type I (BHV-1) expressing rabies virus glycoprotein (RABV G) instead of its gE glycoprotein (gE) by CRISPR-Cas9 and homologous recombination technology (BHV-1-ΔgE-G). Insertion of the RABV G gene is stable after 20 rounds of *in vitro* passaging and the recombinant virus replicates to high titers in MDBK cells. The RABV G expresses in the recombinant virus-infected cells and on the virion surface of BHV-1-ΔgE-G. One single immunization with BHV-1-ΔgE-G-activated dendritic cells (DCs) and B cells furthermore induced a protective immune response in mice against severe lethal challenge infection. A protective level of RABV-specific virus-neutralizing antibody (VNA) was detected in intramuscular immunized mice and cattle without any clinical symptoms. This research demonstrated that the BHV-1 vector-based RABV vaccine is a potential candidate for cattle.

## Introduction

According to the World Health Organization (WHO) estimates, rabies is endemic in more than 150 countries worldwide, and about 59,000 people die from rabies every year (World Health Organization., 2013). Due to the limitation of effective control in animal reservoirs in developing regions, rabies in stray dogs, cattle, goats, camels, and wild animals have increased in the north part of China, provincial regions, where rabies had rarely been reported previously (Shao et al., 2011; Liu et al., 2016; Feng et al., 2020; Li et al., 2020). Although the canine rabies vaccine has been recommended for cattle by the World Organization for Animal Health and successfully performed in rabies endemic countries (Yakobson et al., 2015), it is still costly and uncertain that local rabies vaccine could block the spread of infection in cattle.

Efficacious rabies vaccines are for safety and commercial purposes. Worldwide, human rabies vaccines are based on the fixed strains of RABVs that are grown in cell culture, which contain inactivated virus and adjuvant (DiStefano et al., 2013). Usually, these vaccines need to be given three to five times in a prophylactic immunization to achieve protective immunity (Zhou et al., 2006). On the other hand, SAD-modified live RV vaccines used in wild animals have effectively reduced the incidence of fox-derived rabies in Western Europe (Freuling et al., 2013). However, these recombinant RABV vaccines are still based on the RABVs genome, which raises the possibility of recombination between the vaccine RABVs and wild-type RABVs resulting in unpredictable possibilities.

Rabies virus is a single-stranded negative-strand RNA virus with a total genome length of about 12 kb, encoding five structural proteins: nucleoprotein (N), phosphoprotein (P), matrix protein (M), glycoprotein (G), and large transcription protein (L). Among them, RABV G is a trimeric transmembrane protein, expressed on the surface of the RABV virus, and it has specific recognition sites for B cells and T cells on its surface, which can cause an immune response and is a key protein that induces the production of VNA. Therefore, RABV G protein is often used in the construction of recombinant rabies vaccines. To vaccinate livestock in developing countries, a low-cost vaccine with one injection would be ideal. Live recombinant viral vectors which induced excellent humoral and cellular immunity are considered to be effective vaccine candidates to deliver antigens derived from pathogens. Various vector rabies vaccines have been constructed with different viral vectors, such as Parapoxvirus, Newcastle disease virus, Sindbis virus, parainfluenza virus, herpes virus, adenovirus, and baculovirus. Moreover, a safe and effective rabies vaccine especially for cattle still needs to be further developed.

Bovine herpes virus type I (BHV-1) is a double-stranded DNA virus belonging to the family Herpesviridae, subfamily Aphaherpesvirinae, with a total genome length of about 138 kb, and 10 of the proteins it encodes are envelope glycoproteins. Among them, gE glycoprotein, as a transmembrane glycoprotein, is a non-essential protein for virus replication. The current BHV-1 live vaccine in which gE is deleted has been widely used in Europe, so BHV-1 lacking the gE gene is safe as a vector for constructing recombinant rabies virus (Petrini et al., 2019). A similar construct using BHV type 5 for cattle has been developed to protect the animals in Latin America both against BHV-5 and rabies (bats-transmitted rabies) (Ana et al., 2002). Also, recombinant Canine Herpesviruses expressing RABV G have been tested as immune-contraceptive vaccines for carnivores (Chen et al., 2019). Furthermore, the herpesvirus genome is substantial enough to tolerate the insertion of large foreign genes and have a stable genome and technologies for the construction of recombinants that are well-established. For instance, recombinant BHV-1 or BHV-4 expressed other viral antigens have developed in animals and have good immunogenicity (Schrijver et al., 1997; Kweon et al., 1999; Macchi et al., 2018; Pedrera et al., 2020; Chowdhury et al., 2021). These characteristics above suggest that BHV-1 may be a potential vaccine candidate for preventing rabies in cattle and other ruminants.

Here, we used CRISPR-Cas9 and homologous recombination technology to construct a recombinant BHV-1 expressing RABV G and evaluated the replication of BHV-1-ΔgE-G, the expression of RABV G, genetic stability *in vitro*, attenuation, immune response, and immunogenicity in animal models. Our results show that a single dose of BHV-1-ΔgE-G induces a protective level of RABV VNA in cattle serum and BHV-1-ΔgE-G provides complete protection against the lethal challenge of RABV in mice.

## Materials and Methods

### Cells, Viruses, Antibodies, and Plasmids

Madin–Darby bovine kidney (MDBK) cells, human embryonic kidney 293T (HEK293T), and African green monkey kidney cells (VERO-E6) were kept in our lab and cultured in Dulbecco's modified eagle's medium (DMEM, Gibco, CA, USA) containing 10% fetal bovine serum (FBS, Gibco, CA, USA), and mouse neuroblastoma (NA) cells were maintained in Roswell Park Memorial Institute (RPMI) 1640 medium (Gibco, CA, USA) supplemented with 10% FBS. In brief, DCs isolated from mouse bone marrow and cultured in RPMI 1640 supplemented with 10% FBS and 20 ng/ml granulocyte-macrophage colony-stimulating factor (GM-CSF, PeproTech, USA) for 7 days as previously described (Yang et al., 2015). The parent BHV-1 (named NM14, GenBank No. of gE gene is MK035760.1) was isolated from bovine and stored in our lab as described previously (Mu et al., 2018). Challenge virus standard CVS-24 strain of rabies virus was bred in the sucking mice brain and a neuro attenuated rabies virus SAD-L16 was bred in the NA cells (Morimoto et al., 1999). Fluorescein isothiocyanate (FITC)-conjugated antibodies against the RABV N protein were purchased from FujiRab (Melvin, PA).

### Animals

ICR mice were purchased from Charles River (Beijing Vital River Laboratory Animal Technology Co., Ltd.). Experimental infectious studies were approved by the Ethics Committee of Inner Mongolia University (IMU-MO-2020-031). All healthy male cattle (6–8 months old) were obtained by the pasture, and their BHV-1 antigen and antibody tests were negative. All cattle were divided into three groups and immunized with BHV-1-ΔgE-G, SAD, or DMEM by intramuscular injection, and bovine serum was collected at 1, 2, 3, 6, 12, 18 weeks. The above immunization experiments were carried out entirely with the permission of the rancher. All efforts were made to minimize the suffering of the animals.

### Donor Plasmids and sgRNA CRISPR/Cas9 Plasmids Construction

**Figure 1:**
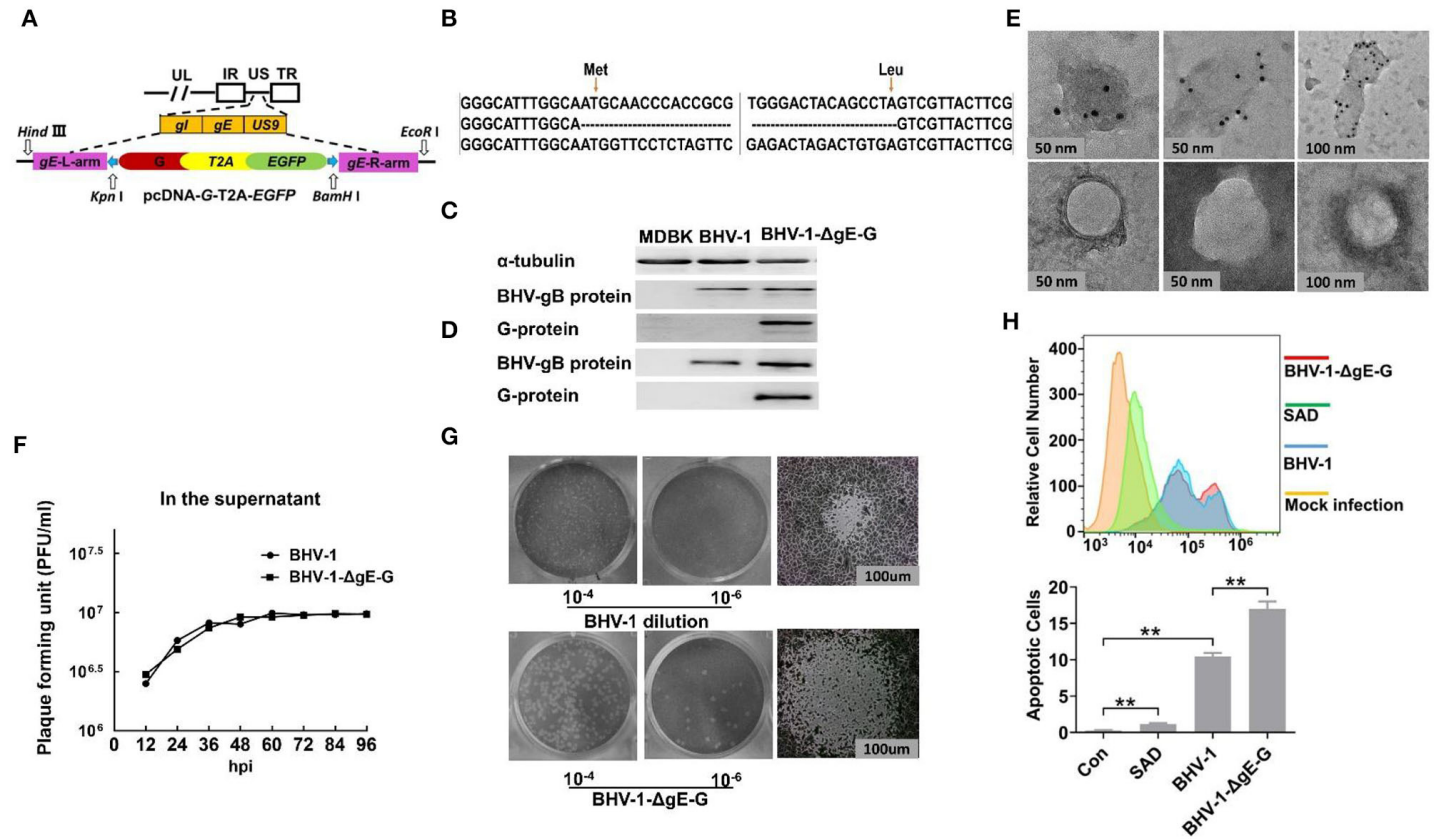
Construction and characterization of BHV-1-ΔgE-G. **(A)** Schematic on the generation of a recombinant viral vector. The pcDNA3.1 expression vector is used as a backbone to construct the donor template. gE homologous arms were amplified using PCR from BHV-1 with the primer pairs L-ge and R-ge, the RABV G gene was amplified using PCR from SAD with primer pairs G-F and G-R, and the T2A and EGFP genes were amplified using PCR from pCAG-EGFP with primer pairs T2A-EGFP-F and T2A-EGFP-R. These PCR products were digested with enzyme sets HindIII & EcoR I, Kpn I & Not I, and Not I & BamH I, and then ligated into pcDNA3.1 plasmid vector (pcDNA-LgE-G-EGFP-RgE), respectively. **(B)** Viral genome DNA isolated from BHV-1-ΔgE-G infected MDBK cells was used to carry out PCR and sequencing. **(C)** The RABV G expressed in MDBK infected with BHV-1-ΔgE-G at 24 hpi was detected by Western blot. **(D)** The RABV G incorporated in virion of BHV-1-ΔgE-G was purified by sucrose gradient ultracentrifuge and detected by Western blot. **(E)** The RABV G incorporated in virion of BHV-1-ΔgE-G was purified by sucrose gradient ultracentrifuge and analyzed by EM. **(F)** Multistep growth curves of BHV-1-ΔgE-G and BHV-1 in MDBK cells. MDBK cells were infected with the indicated viruses at an MOI of 0.01, and the virus titers in infected cell supernatants were determined at indicated time point. **(G)** The plaque diameter of BHV-1-ΔgE-G and BHV-1 was analyzed by the viral plaque assay using a Nikon ECLIPSE TS100 microscope. **(H)** The prorogation of apoptotic cells infected by BHV-1-ΔgE-G, BHV-1, and RABV was determined with BD Accuri C6 Flow Cytometer, and the data were analyzed with FlowJo software (TreeStar, San Carlos, CA). Significance was assessed using Student's *t*-test. **P ≤ 0.01.

Figure 1A is a schematic of the generation of the recombinant viral vector. sgRNAs were designed using the online CRISPR Design Tool (https://zlab.bio/guide-design-resources), and the guide RNAs were cloned into the pSpCas9(BB)-2A-Puro (PX459) V2.0 vector (Addgene, Cambridge, MA, USA, Plasmid #62988) as previously described (Cong et al., 2013). All primers are used in the present study list in Table 1. The primer pairs of gE-sgRNA1-F/gE-sgRNA1-R and gE-sgRNA2-F/gE-sgRNA2-R were targeted to gE of BHV-1, and the primer pairs of EGFP-sgRNA1-F/EGFP-sgRNA1-R and EGFP-sgRNA2-F/EGFP-sgRNA2-R were targeted to EGFP to delete EGFP from EGFP positive recombinant BHV-1. The pcDNA3.1 (+) expression vector (Clontech, PaloAlto, CA, USA) was used as a backbone to construct the donor template. The gE homologous arms were amplified using PCR from BHV-1 with left homologous arms primer pairs (LgE-F and LgE-R) and right homologous arms primer pairs (RgE-F and RgE-R), the RABV G gene was amplified using PCR from SAD-L16 with primer pairs G-F and G-R, and the T2A and EGFP gene were amplified using PCR from pCAG-EGFP (Addgene, Cambridge, MA, USA, Plasmid #89684) with primer pairs T2A-EGFP-F and T2A-EGFP-R. These PCR products were digested with the enzyme sets HindIII & EcoR I, Kpn I & Not I, and Not I & BamH I, and then ligated into pcDNA3.1 plasmid vector (pcDNA-LgE-G-EGFP-RgE), respectively. The pcDNA-LgE-G-EGFP-RgE plasmid was verified by sequencing with primer pairs (BHV-1-gE-F and BHV-1-gE-R).

**Table 1:** Primers used in the study.

**Primer**	**Sequence (5** ^**′**^ **to 3** ^**′**^ **)**	**Restriction enzyme sites**
gE-sgRNA1-F	CACCGCGAGCCCGGGGTTTCGGTCGCGG	BbsI
gE-sgRNA1-R	AAACCCGCGACCGAAACCCCGGGCTCGC	BbsI
gE-sgRNA2-F	CACCGCCACGTCGGTGAAGCACTCGCGG	BbsI
gE-sgRNA2-R	AAACCCGCGAGTGCTTCACCGACGTGGC	BbsI
EGFP-sgRNA1-F	CACCGGTCGCCCTCGAACTTCACCT	BbsI
EGFP-sgRNA1-R	AAACAGGTGAAGTTCGAGGGCGACC	BbsI
EGFP-sgRNA2-F	CACCGGTTGGGGTCTTTGCTCAGGG	BbsI
EGFP-sgRNA2-R	AAACCCCTGAGCAAAGACCCCAACC	BbsI
G-F	CGGGGTACCTCTTGGATGTGAAAAAAACTATTAAC	KpnI
G-R	AAGGAAAAAAGCGGCCGCCAGTCTGGTCTCACCCCCACTCTTG	NotI
T2A-EGFP-F	AAGGAAAAAAGCGGCCGCGCAAAAAAGAAAAAGGA	NotI
T2A-EGFP-R	CTAGGGATCCGCAACTAGAAGGCACAG	BamHI
LgE- F	CGAAAGCTTTCGCCTCCTGCCCGCG	HindIII
LgE-R	TTTGGTACCCTCTCGCGTGCGC	KpnI
RgE-F	AAAGGATCCAGTCGTTACTTCGGACCGTTTGGTG	BamHI
RgE-R	AAGGAATTCCAGCGCCTCGATAGTTTTCGTTGAC	EcoRI
BHV-1-gE-F	CGCCGGGTTGTTAAATGGGTCTCG	_
BHV-1-gE-R	GGGGCGCGTCCTCGATGGTG	_

*Underlined are restriction sites*.

### Virus Recombination and Plaque Purification

The sgRNA/Cas9 vectors and donor vectors were co-transfected into VERO-E6 cells with 1 μg of sgRNA/Cas9 plasmid and 4 μg of pcDNA-LgE-G-EGFP-RgE plasmid using Lipofectamine 3000 (Invitrogen, CA, USA) according to the manufacturer's instructions. After 6-h transfection, the parent BHV-1 was infected at a multiplicity of infection (MOI) of 0.01 and the culture medium was changed to DMEM with 2% FBS, and cells were collected at 48-h post-infection (hpi). After three rounds of freeze and thaw, the cell lysate was centrifuged for 5 min at 10,000 rpm/min. The supernatant was used for the next propagation or stored at −80°C. The first-generation recombinant virus was purified by fluorescence-activated cell sorting. For recombinant virus purification, the infected MDBK cells (MOI = 0.01) were covered by 1% low melting-point agarose. After 72 h of infection, well-separated plaques with positive EGFP or negative EGFP were picked up by pipette tip and stored in microtubes containing 200 μl serum-free DMEM. The rescued virus was subjected to at least 10 rounds of plaque purification assay. All plaque purified viruses were passaged 20 rounds on MDBK cells and then evaluated by PCR, flow cytometry, viral plaque assay, and Western blot as described below.

### Purification of Recombinant BHV-1 by Fluorescence-Activated Cell Sorting

The fluorescence-activated cell sorting was performed as previously described with slight modifications (Di Lullo et al., 2010). The fluorescence-activated cell sorting was performed on BD FACSAria cell sorter (BD Biosciences, USA) with 488 nm lasers. MDBK cells were infected with the first-generation recombinant BHV-1 at an MOI of 0.01. At 48 hpi, cells were washed, trypsinized, washed again, resuspended in a culture medium, and kept on ice. The MDBK cells expressing EGFP were sorted into a 96-well plate with 5,000 cells per well.

### Plaque Assay for BHV-1

BHV-1 was titrated on MDBK cells by viral titering-Plaque Assay. MDBK cells were cultured in 12-well plates for 12 h and then infected with 10-fold dilutions of BHV-1. After 2 h of incubation, cells were washed with PBS and then cultured in DMEM with 1% FBS and 4% sodium carboxymethyl cellulose (Sigma, USA). After 2 days of incubation at 37°C, cells were fixed with 10% formaldehyde and stained with crystal violet. The number of plaques was recorded and the plaque-forming unit per ml (PFU/mL) was calculated as follows: Virus titer (PFU/mL) = (number of plaques per well × virus dilution factor)/virus inoculation per mL).

### Western Blotting

MDBK cells extract or purified virions were lysed in hot Laemmli sample buffer (BIO-RAD, USA) and boiled for 5 min. All samples were resolved by 10% sodium dodecyl sulfate-polyacrylamide gel electrophoresis (SDS-PAGE) and transferred onto a polyvinylidene difluoride (PVDF) membrane. PVDF blocked with tris-buffered saline (TBS) contained 1% bovine serum albumin (BSA) and then incubated with respective primary antibodies at room temperature for 2 h, followed by horseradish peroxidase-conjugated secondary antibodies (Sigma) for 1 h at room temperature. Proteins were detected by west pico chemiluminescent substrate (Thermo Fisher Scientific, United States). Band signals corresponding to immunoreactive proteins were developed by exposure in the Tanon 5200 imaging system (Tanon Science and Technology, Beijing, China) using Tanon MP software. An anti-BHV-1 gB antibody was purchased from VMRD INC. (WA, USA) and anti-RABV G (G53) monoclonal antibody was prepared as previously described (Jiang et al., 2010).

### Electron Microscopy and Immunogold Labeling for BHV-1-ΔgE-G

Viruses are purified by 3K Ultra-15 Centrifugal Filter Units (Millipore, USA). The G in BHV-1-ΔgE-G was labeled with 10-nm immunogold and evaluated by transmission electron microscopy (TEM) as described previously with minor modification (Li et al., 2019). In brief, viruses were absorbed onto parlodion-coated nickel grids for 30 min followed by fixation with 2.5% glutaraldehyde for 30 min. Grids were then washed with a drop of TBS, pH 7.4, three times for 5 min, followed by floating on a drop of RABV G-specific monoclonal antibody (G53) diluted to 1:300 in TBS containing 1% BSA for 1 h. After washing with TBS three times, samples were incubated for 1 h with Goat Anti-Mouse IgG H&L (10 nm Gold), and preadsorbed (Abcam, USA) and diluted at 1:10 in TBS containing 1% BSA. Grids were again washed with TBS and then stained with 2% phosphotungstic acid (pH 7.0) for 30 s. The grids were then observed under an FEI Tecnai G^2^ F20 S-Twin transmission electron microscope (Thermo Fisher, USA).

### Flow Cytometry

For immune cells activation analysis, inguinal lymph nodes of mice were taken out after administration and slowly added to a 100-μm cell filter (Sigma-Aldrich) with a pipette gun for filtration and centrifuged the obtained filtrate at room temperature at 400 g for 5 min. Samples were resuspended with PBS buffer containing 2% FBS and 20 nM EDTA. The above steps were repeated twice. Cells were stained with fluorescent-labeled differential markers CD4, CD11c, CD86, CD80, and CD138, major histocompatibility complex class II (MHC-II), IL-17, Foxp3 antibodies, and an isotype control for 1 h and washed twice, then measured by flow cytometry. All the antibodies used in the flow cytometry above were purchased from BD biosciences (USA). For apoptosis assay, MDBK cells were infected with BHV-1, BHV-1-ΔgE-G, or SAD virus for 48 h at MOI of 0.01, respectively. Cells were collected at 48 hpi and detected the apoptosis using FITC Annexin V Apoptosis Detection Kit I (BD biosciences, USA) by flow cytometry. Cells were suspended with PBS containing 1% FBS and 2 μM EDTA solutions, washed twice with cold PBS, and resuspended cells in 1X Binding Buffer at a concentration of 10^6^ cells/ml. Transfer 100 μl of the solution (1 × 10^5^ cells) to a 1.5-ml centrifuge tube. Add 5 μl of FITC Annexin V and 5 μl PI to cells undergoing apoptosis (FITC Annexin V positive and PI negative). Gently vortex the cells and incubate for 15 min at RT (25°C) in the dark. Add 400 μl of 1X Binding Buffer to each tube and analyze by flow cytometry within 1 h. Flow cytometry was performed on BD AccuriTM C6 flow cytometry (BD Bioscience), and data were analyzed by BD FACSDiva (BD Pharmingen) and FlowJo software (TreeStar, San Carlos, CA), and finally, GraphPad Prism 5.0 software was used to plot the analyzed data.

### Statistics Analysis

Statistical significance of the differences between groups was determined using student's *t*-test with ^***^ indicating a *p* < 0.001, ^**^ a *p* < 0.01, and ^*^a *p* < 0.05 using Graph Pad prism software (GraphPad Software, Inc., CA). The statistical significance of survival rates was determined by the log-rank test and Kaplan–Meier survival analysis.

### Ethics Statement

All animal procedures were approved by the Ethics Committee of Inner Mongolia University (IMU-MO-2020-031). All efforts were made to minimize animal suffering. The animal experiments involving the infection with RABV were carried out in the animal facility with ABSL-2 level at Inner Mongolia University.

## Results

### Construction and Characterization of Recombinant BHV-1 Expressing RABV G

Our lab's previous report indicated that EGFP inserted between the gI and US9 genes of BHV-1 was expressed well in MDBK cells (Mu et al., 2018). The safety and immunogenicity of recombinant BHV-1 have also been approved in the last decades (Kaashoek et al., 1994). To explore the potential of BHV-1 as a live prophylactic vaccination candidate for protection from rabies, the RABV G gene was used instead of the partial gE gene (amino acid position of gE, 136 Met to 513 Leu) for constructing a recombinant BHV-1. To avoid false genetic recombination caused by green fluorescent gene expression in the homologous recombination donor template, the expressions of RABV G and EGFP were driven by the endogenous viral promoter without other specific promoters, which occurs only after precise DNA homologous recombination (Figure 1A). The recombinant virus, BHV-1-ΔgE-G, was established with two steps of CRISPR/Cas9 and DNA homologous recombination and purified with FACS technique as specifically described in Materials and Methods section. First, the gE gene of BHV-1 was replaced with RABV G and EGFP by CRISPR Cas9-mediated homologous recombination. Next, the EGFP in the recombinant was deleted by CRISPR Cas9-mediated homologous recombination. After 20 rounds of virus passage in the MDBK cells, the stability of the inserted gene was confirmed by PCR analysis and sequencing (Figure 1B). Expression of the G protein was detected by Western blot in BHV-1-ΔgE-G-infected MDBK cells. At 24 hpi, specific expression of RABV G in MDBK was detectable (Figure 1C). The BHV-1-ΔgE-G was purified by sucrose gradient ultracentrifuge and the G incorporated into the virions was analyzed by Western blot (Figure 1D). A RABV G band was detected in the BHV-1-ΔgE-G virions, whereas no RABV G band was found in the parental BHV-1 virions. To visually observe the expression of RABV G on BHV-1-ΔgE-G, the virus particles of BHV-1-ΔgE-G were analyzed using RABV G-specific antibodies and examined with an electron microscope (EM). RABV G was detected on the surface of BHV-1-ΔgE-G which indicated that RABV G is incorporated into the recombinant BHV-1 particles (Figure 1E).

MDBK cells were infected with parental BHV-1 or BHV-1-ΔgE-G at an MOI of 0.01, and the supernatants were quantified by viral plaque assay in MDBK cells. As shown in Figure 1F, BHV-1-ΔgE-G had similar initial growth kinetics, and both the virus titers of parental and recombinant BHV-1 reach 10^6.5^ FFU at 48 hpi. The viral plaque morphology was also observed under the microscope. The plaque diameter of BHV-1-ΔgE-G was almost twice the diameter of parental BHV-1 (Figure 1G). Previous studies have reported that the RABV G induces cell apoptosis (Sarmento et al., 2005). To investigate whether the stronger cytopathic effect (CPE) of BHV-1-ΔgE-G is induced by the apoptosis, MDBK cells were infected with BHV-1, BHV-1-ΔgE-G, or SAD for 48 h at an MOI of 0.01, respectively (Figure 1H). The prorogation of apoptotic cells infected by BHV-1-ΔgE-G (17.0%) was higher than BHV-1 (10.4%) and RABV (1.2%).

### Clinical Observations and VNA Persistence of BHV-1-ΔgE-G in Mice

**Figure 2:**
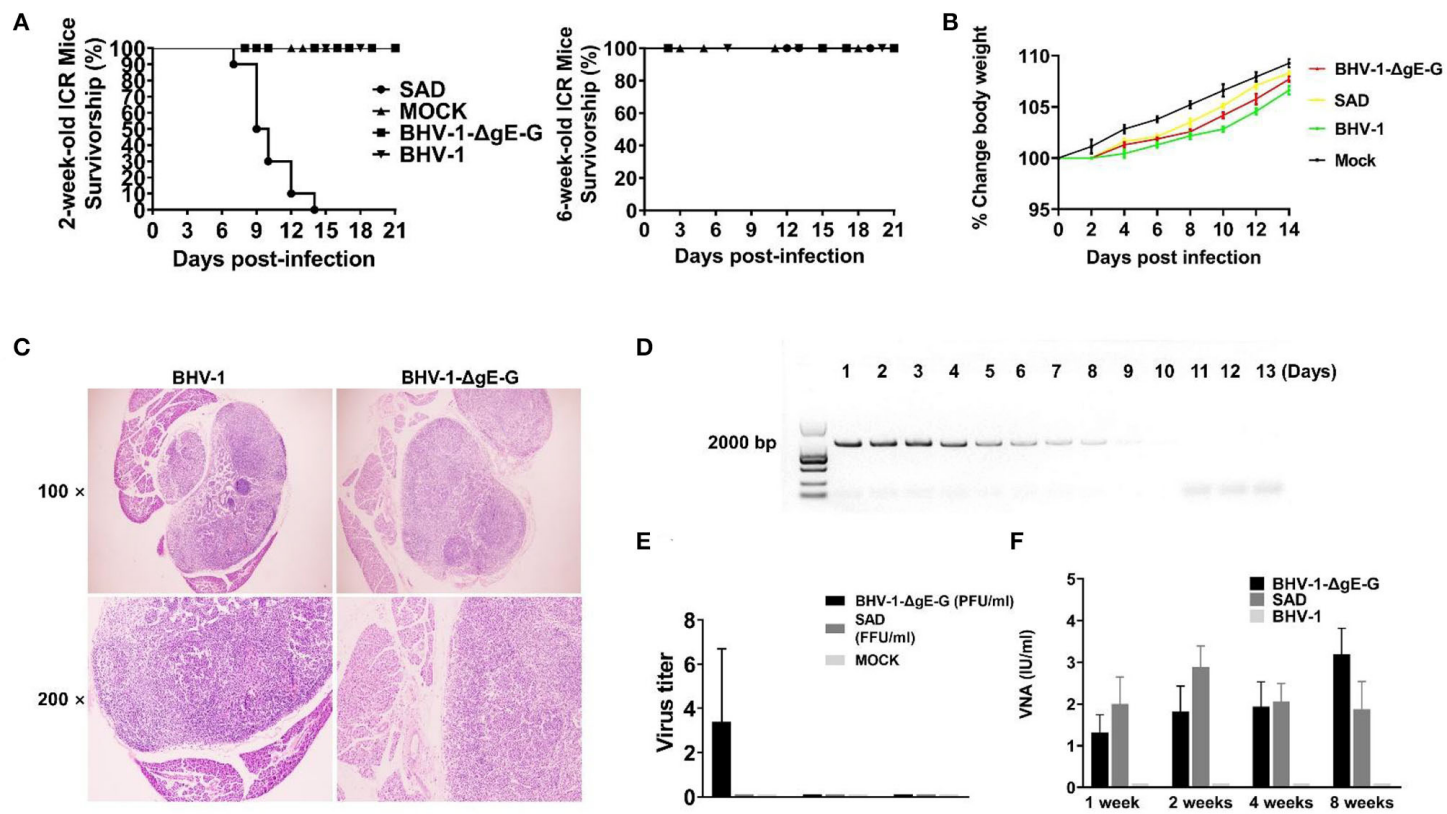
Clinical observations and VNA persistence in mice that administrated SAD and BHV-1-ΔgE-G via the IM route. **(A)** The survivorship of 2-week-old and 6-week-old female ICR mice inoculated with the indicated virus via IM route. **(B)** Body weight change (%) was evaluated in mice which were injected with the indicated virus via the IM route. **(C)** Inguinal lymph nodes were obtained from immunized mice and subjected to HE staining. **(D)** The gB gene expression in mice inguinal lymph nodes that were immunized with BHV-1-ΔgE-G was detected by PCR and analyzed with electrophoresis. **(E)** The inguinal lymph nodes were obtained from mice immunized with the indicated virus and evaluated the virus titer. **(F)** The serum of mice inoculated with the indicated virus was subjected to RFFIT for the analysis of VNA.

To estimate any adverse effects on mice that might induce by BHV-1 infection, 2-week-old ICR mice and 6-week-old female ICR mice were administered with 10^6^ FFU of BHV-1, BHV-1-ΔgE-G, SAD, or mock-infected with DMEM via intramuscularly (IM) route. All the 2-week-old mice injected with SAD succumbed to infection within 15 days. In contrast, all mice infected with BHV-1-ΔgE-G or BHV-1 were non-lethal mice (Figure 2A). As previously reported, the SAD and its derivatives remained pathogenic for young rodents (Rasalingam et al., 2005).

For 6-week-old mice, RABVs were non-pathogenic. All mice (*n* = 10) were observed daily for 2 weeks with body weight change and clinical symptoms. After 4 days of immunization, the body weight of all mice increased gradually as normal mice (Figure 2B). None of the clinical symptoms were observed in the mice after administration with any of the viruses mentioned above. To determine whether the BHV-1 infection in mice was reflected in the pathological change of inguinal lymph nodes, surrounding tissues were collected from mice for histopathology. There was no significant histological change between BHV-1-ΔgE-G immunized mice and mock-immunized mice (Figure 2C). The gB of BHV-1 can be detected from 1 day to 9 days after immunization in mice inguinal lymph nodes, and the expression level of gB was decreased from 5 days post-immunization (Figure 2D). The live BHV-1-ΔgE-G in mice inguinal lymph nodes can be detected within 1 week and the virus titer was lower than 10 PFU/ml (Figure 2E). To evaluate the persistence of the VNA against RABV, the mice sera were collected at 1, 2, 4, and 8 weeks after IM immunization and the VNA in serum was estimated by the fluorescent antibody virus neutralization (FAVN) test. As expected, BHV-1-ΔgE-G induced a persistent and high level of VNA in mice serum, 1 week after immunization until 8 weeks, and the VNA level was higher than 1 IU (Figure 2F). The VNA level of BHV-1-ΔgE-G immunized mice was similar to the SAD-immunized mice, both of which were higher than 0.5 IU, which was the minimum protective antibody level in carnivores (Cliquet et al., 1998).

### BHV-1-ΔgE-G Enhances the Activation of DC and B Cell

**Figure 3:**
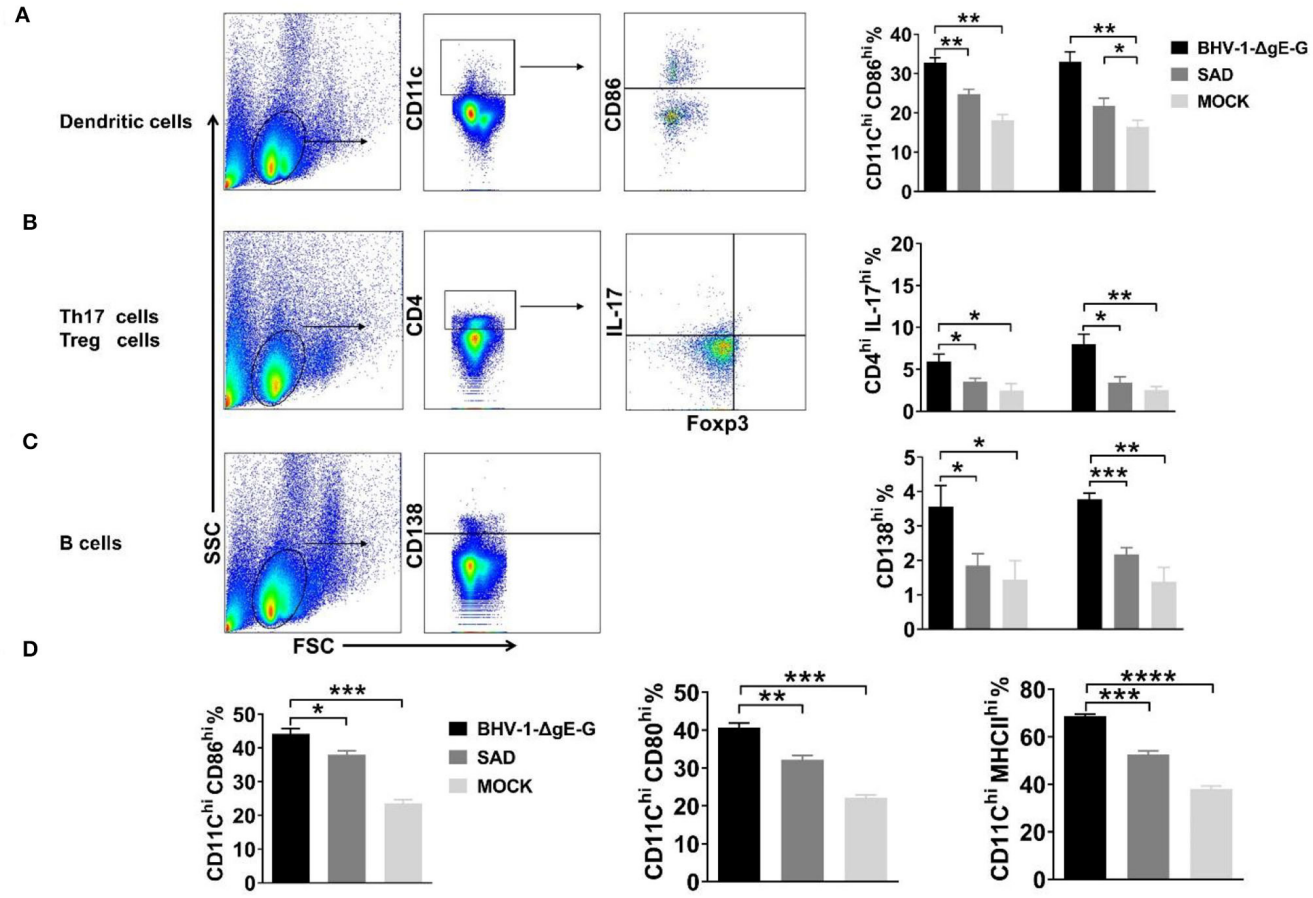
BHV-1-ΔgE-G induces early activation of immune cells. **(A–C)** Flow cytometric analysis of activation status of DCs, Th 17 cells, and B cells in mice inguinal lymph nodes which were immunized with the indicated virus via the IM route. **(D)** Flow cytometric analysis of CD11c, CD86, or MHCII expression level of DCs infected with 0.1 MOI of indicated virus at 48 hpi. Significance was assessed using Student's *t*-test. *P ≤ 0.05; **P ≤ 0.01; ***P ≤ 0.001; ****P ≤ 0.0001.

To determine the immune effect of recombinant virus BHV-1-ΔgE-G, 6-week-old ICR mice were immunized via IM route with 2 × 10^5^ PFU of BHV-1-ΔgE-G, 2 × 10^5^ FFU of SAD, or mock-immunized control group (DMEM), respectively. Seven days and 14 days post-immunization, single-cell suspensions were prepared from inguinal lymph nodes of mice, and activation of DCs, Th17 cells, and B cells in lymph nodes were analyzed by flow cytometry. As shown in Figure 3A, the activation of DC cells (CD11c high expression and CD86 high expression) in the lymph nodes of mice which were immunized with the BHV-1-ΔgE-G were significantly higher than SAD and mock-immunized group. In addition, the number of Th17 cells and B lymphocytes in the recombinant virus BHV-1-ΔgE-G group increased significantly, which was higher than that in SAD and mock-immunized groups, indicating that the recombinant virus promoted the increase of Th17 cells and B lymphocytes and activated humoral immunity (Figures 3B,C). To further confirm the activation of DC, whether it is directly induced by the BHV-1-ΔgE-G, the bmDCs were infected with 0.1 MOI of BHV-1, 0.1 MOI of BHV-1-ΔgE-G, 0.1 MOI of SAD, or mock-infected control group (DMEM), respectively. At 48 hpi, DCs were evaluated by FACS, and the number of CD11c^hi^ & CD86^hi^ cells, CD11c^hi^ & CD80^hi^ cells, and CD11c^hi^ & MHC II^hi^ cells in BHV-1-ΔgE-G-infected group were significantly higher than other groups (Figure 3D). These results suggest that the recombinant virus BHV-1-ΔgE-G can activate the DCs *in vivo* and *in vitro*, and further maturated T cells and B cells.

### Induction of Protective Immune Response in Mice

**Figure 4:**
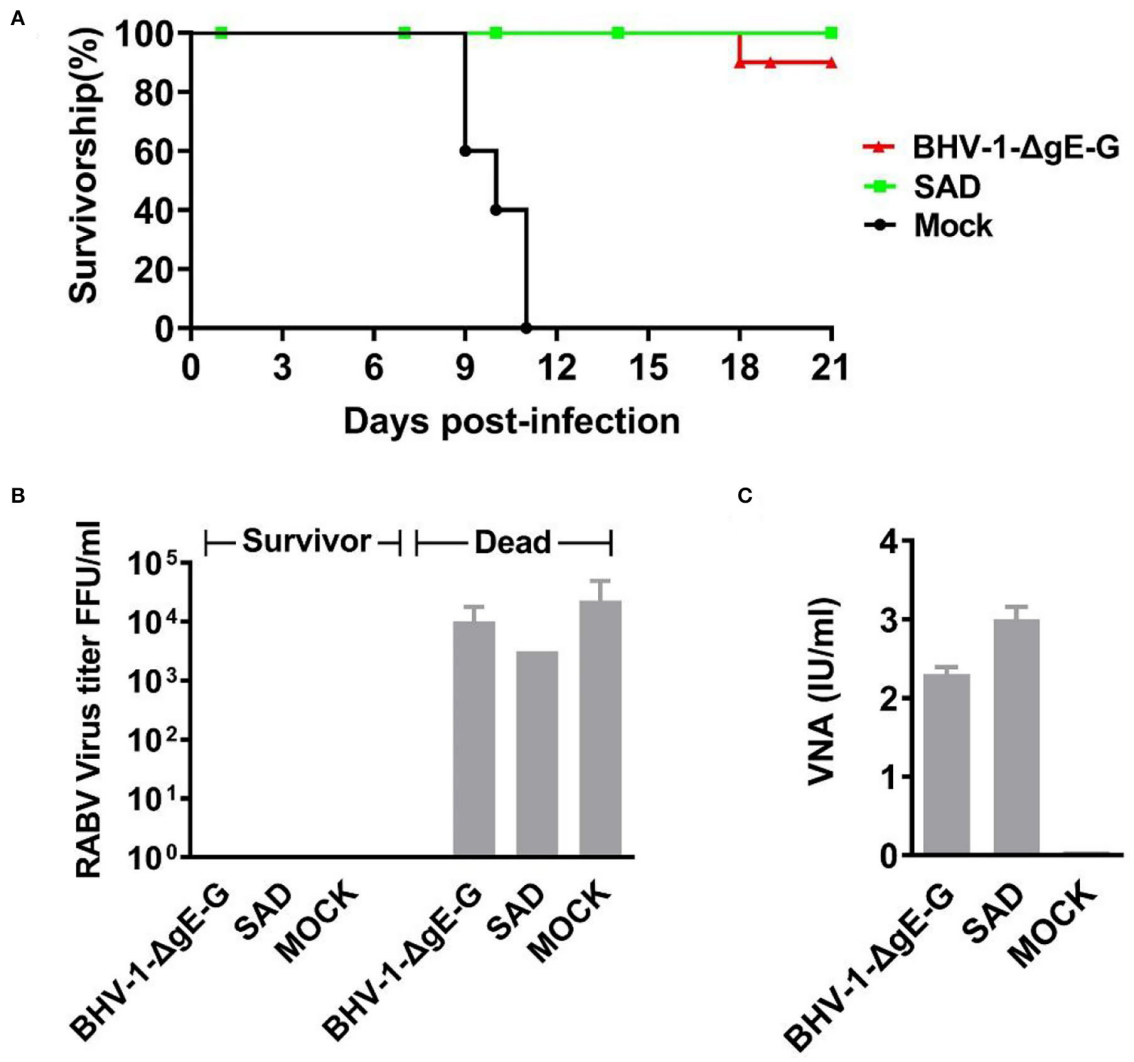
Survival rate and VNA of mice post-challenge. **(A)** Six-week-old female ICR mice were randomly divided into three groups, and each group included 10 mice. Each group of mice was inoculated once with 10^5^ PFU of BHV-1-ΔgE-G, 10^5^ FFU of SAD or medium control, respectively. Each mice was inoculated with 20 μl of virus, 10 μl for each hind leg. All mice received a challenge with 50 LD50 CVS-24 via an IM route at 21 days post-immunization. Mice were seen to be moribund or that had lost more than 30% of their starting body weight were humanely euthanized. The number of euthanized mice and the corresponding days of death were observed, and the results were graphed by GraphPad Prism 5.0 software. **(B)** Virus titers in the brains of surviving and dead mice. **(C)** VNA in the serum of dead and surviving mice.

To determine the immune protection rate of BHV-1-ΔgE-G via IM route, mice were immunized with 2 × 10^5^ PFU of BHV-1-ΔgE-G and 2 × 10^5^ FFU of SAD virus on days 1 and 14, respectively, and DMEM was injected as the mock immunization group. Seven days post the boost, rabies virus Challenge Virus Standard (CVS-24) with 50 of LD50 was used to infect the above mice. The clinical symptoms and survival rate of the mice were observed until 21 days post-infection. As shown in Figure 4A, the survival rates of mice injected with BHV-1-ΔgE-G and SAD were 90 and 100%, respectively. While in the mock-immunization group, all mice died within 11 days after CVS-24 challenge. These results indicate that the recombinant virus BHV-1-ΔgE-G can produce effective immune protection efficiency in mice by intramuscular injection.

In mice that succumbed to rabies, an average virus titer in mice brain reached 10^4.5^ FFU/ml, while the virus titer in mice brain that protected by vaccine is nearly to 0 FFU/ml (Figure 4B). In mice immunized with BHV-1-ΔgE-G or SAD, the average VNA production of 2.25 IU/ml or 3.00 IU/ml was detected, respectively (Figure 4C). Therefore, the immune protection efficiency of BHV-1-ΔgE-G group was same as the SAD, and the recombinant virus BHV-1-ΔgE-G is an ideal vaccine candidate.

### Clinical Observations and Antibody Response of Cattle Immunized With BHV-1-ΔgE-G *via* IM Route

**Figure 5:**
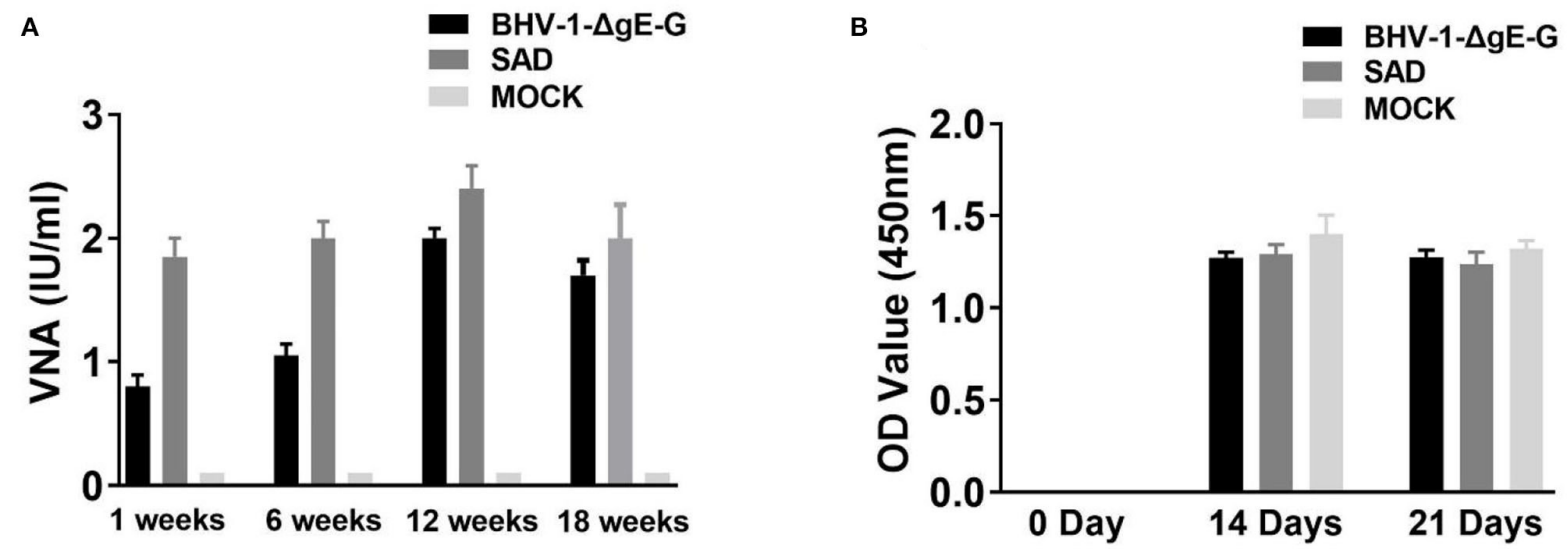
VNA level of cattle immunized with BHV-1-ΔgE-G via IM route. **(A)** Five cattle immunized with a single dose of 10^7^ PFU of BHV-1-ΔgE-G, 10^7^ PFU of SAD, or DMEM. Serum samples were collected at 1, 6, 12, and 18 weeks and subjected RFFIT for VNA analysis. **(B)** Serum samples were collected on 0 day, 14 days, and 21 days and performed ELISA for gB antibody for BHV-1.

To test whether the BHV-1-ΔgE-G is a potential vaccine candidate in cattle, we test the safety, virus persistence period, and RABV-specific VNA in cattles' serum after IM injection. Each group of five cattle was immunized via IM route with a single dose of 10^7^ PFU of BHV-1-ΔgE-G or 10^7^ FFU of SAD virus on day 1, and DMEM was taken as a control group. Clinical symptoms and VNA in cattle serum were observed after being immunized for another 12 weeks without challenge. None of these cattle developed any clinical signs during the observation period. Serum samples were collected at 1, 6, 12, and 18 weeks and subjected to virus titration and VNA analysis. In addition, none of the live SAD and BHV-1-ΔgE-G in cattle's serum was detected within 18 weeks. As shown in Figure 5, both BHV-1-ΔgE-G and SAD vaccinated cattle produce productive serum VNA. The VNA titers of BHV-1-ΔgE-G immunized cattle were 0.8, 1.05, 2.0, and 1.7 IU/ml at 1-, 6-, 12-, and 18-weeks post-immunization, and the VNA titers of SAD-vaccinated cattle were 1.85, 2.0, 2.4, and 2.0 IU/ml at 1-, 6-, 12-, and 18-weeks post-immunization, respectively. Furthermore, to test whether the recombinant virus produced specific immunity to BHV-1, bovine serum was collected on the 14th and 21st days after IM injection, and OD values of gB specific antibodies were detected in the serum by gB antibody ELISA kit (IDEXX, USA). As shown in Figure 5B, OD values of BHV-1-ΔgE-G immunized cattle were 1.273 and 1.276 on 14- and 21-days post-immunization, and OD values of BHV-1-ΔgE-G-immunized cattle were 1.291 and 1.238 on 14- and 21-days post-immunization, indicating that the recombinant virus could produce specific immunity to BHV-1.

## Discussion

The existing rabies vaccine used in animals still has several disadvantages to be improved, such as, reducing the cost of vaccinations, increasing cellular immune response, prolonging the immune protection period, distinguishing wild-type virus infection from vaccine immunization, and decreasing potential pathogenicity. There are four possible ways to further improve the above problems. First, combining several antigens into a multivalent vaccine is a traditional approach used to provide broad coverage of protection for different diseases and reduce the cost of immunization. Second, for the development of immune responses specific to RABV infection, our lab previously constructed several recombinant RABVs that expressed cytokine or chemokine to enhance innate and adaptive immune responses (Wen et al., 2011; Li et al., 2017). Third, the risk of pathogenicity enhancement in RABV-based live rabies vaccines used in animals needs to be seriously considered (Hostnik et al., 2014; Vuta et al., 2016). A recombinant virus vector expressed that RABV G might be a possible way to solve this problem. And several other virus-based vectors may also induce a higher and long-lasting immunoreaction. Fourth, due to the live-rabies virus used in wildlife, any RABV isolated from animals in a vaccination region needs a rapid characterized method using monoclonal antibodies or molecular techniques to distinguish vaccine RABVs from wild-type RABV. A recombinant rabies vaccine containing molecular markers would be a good solution.

In the present study, BHV-1 was shown to induce a rapid and stronger immune response by recruiting and activating DCs, which also maturate B cells and result in secreting of VNA against RABV. IM vaccination of BHV-1-ΔgE-G induced a protective level of VNA response in mice and cattle. Furthermore, a single dose of BHV-1-ΔgE-G could protect mice from 50 LD50 of CVS-24 RABV challenge, and IM-immunized cattle developed a RABV-specific serum antibody titers higher than 0.5 IU/ml within 14 days. A key characteristic of herpes virus infection is that they persist and induce durable immune responses in their infected hosts. Previous studies reported that replication-defective herpes viruses have been used as vectors for gene therapy (Burton et al., 2005) or for vaccine, including Simian immunodeficiency virus (SIV) (Kaur et al., 2007), Influenza virus, Dengue virus (DENV) (Bischof et al., 2017), Porcine circovirus type 2 (PCV2) virus (Chi et al., 2014), Bovine viral diarrhea virus (BVDV) (Chowdhury et al., 2021), Bovine respiratory syncytial virus (BRSV) (Schrijver et al., 1997), Peste des petits ruminants virus (PPRV) (Macchi et al., 2018), and Nipah Virus (Pedrera et al., 2020). Furthermore, Canine Herpesviruses and BHV-5 have been validated safety and effective for a rabies vaccine vector. On the other hand, cellular apoptosis can activate innate and adaptive immunity that may prevent and treat infectious diseases and cancer (Restifo, 2000). As well, large numbers of apoptotic cells entirely triggered DC maturation and processed intracellular antigens from apoptotic cells (Rovere et al., 1998). Previous studies reported that apoptosis in rabies may block virus replication and apoptosis in inflammatory cells and promote the elimination of the virus by the host inflammatory response (Suja et al., 2011). In our study, the G of BHV-1-ΔgE-G was specifically expressed intracellular and on the virion. Moreover, the BHV-1-ΔgE-G induced a stronger apoptosis response than parent BHV-1 or RABV. In particular, almost all cattle will be immunized with the BHV-1 vaccine in most areas of China, especially in the grazing areas where the BHV-1-ΔgE-G will also effectively block rabies transmitted by wild animals. Meanwhile, it can reduce the cost and inconvenience of reimmunization against rabies.

The safety of the novel vaccine vector is considered the most important feature, but it is hard to evaluate the vaccine in all nature host animals. Therefore, we examined the safety of BHV-1-ΔgE-G in cells, mice, and cattle. The RABV G gene was inserted between the gI and US9 genes of BHV-1 strain NM14 and was correctly expressed in good amounts, which did not detectably alter the growth characteristics of parental BHV-1, and the RABV G was stably inherited after 20 rounds of passage in cells, as similarly reported after expression of BVDV envelope protein and PPRV envelope glycoprotein in a BHV-4 vector (Kweon et al., 1999; Macchi et al., 2018). Furthermore, in immunized mice that are non-permissive for BHV-1, only early BHV-1 genes are expressed, and live BHV-1 cannot detect after 14 days inoculation in blood of mice and consequently cannot induce efficient anti-BHV-1 specific immunity. Previous research reported that a replication-deficient HSV-1 vector also provided a good immune response (Samaniego et al., 1998); hence, we will construct a replication-deficient BHV-1 vaccine vector to enhance the safety in the next step of the experiment.

Previous herpes virus-based vaccine was constructed by homologous recombination, which requires to pick hundreds of virus plaques to get one recombinant virus. We combined highly efficient DNA virus recombinant systems in BHV-1 construction, CRISPR/Cas9, and single cell FACS technology to increase the viral gene editing efficiency and virus purification efficiency. Our previous study also reported that BHV-1 gene recombination efficiency in VERO-E6 cells is significantly higher than other cells and using a pair of gRNAs can promote homologous recombination efficiency. Therefore, we can obtain recombinant BHV-1 by a simple plaque purification procedure.

In the present study, we constructed a recombinant BHV-1 that expressed RABV G (BHV-1-ΔgE-G) using CRISPR-Cas9 and estimate the safety and immunogenicity as a multivalent vaccine against animals' rabies in cattle. Animal experiments demonstrated that BHV-1 did not cause any adverse clinical syndromes in mice and cattle, and this vaccine might have a potential to control the cattle and other ruminants derived rabies epidemic in China and some developing countries.

## Data Availability Statement

The original contributions presented in the study are included in the article/Supplementary Material, further inquiries can be directed to the corresponding authors.

## Ethics Statement

The animal study was reviewed and approved by Ethics Committee of Inner Mongolia University (IMU-MO-2020-031). Written informed consent was obtained from the owners for the participation of their animals in this study.

## Author Contributions

YY and WH: writing—review and editing. YY and JG: writing—original draft preparation. CZ: methodology. JG: supervision. LJ and HQ: data curation. YW: visualization. All authors have read and agreed to the published version of the manuscript.

## Funding

This research was funded by the National Natural Science Foundation of China (Grant 32072900 to YY), the Program for Young Talents of Science and Technology in Universities of Inner Mongolia Autonomous Region (No. NJYT22106 to YY), the Science and Technology Major Project of Inner Mongolia Autonomous Region of China (Nos. 2020ZD0008, 2021ZD0013, and 2021ZD0048), and the High-Level Talent Scientific Research Startup Fund for Baotou Teachers' College (No: BTTCRCQD2020-004 to CZ).

## Conflict of Interest

The authors declare that the research was conducted in the absence of any commercial or financial relationships that could be construed as a potential conflict of interest.

## Publisher's Note

All claims expressed in this article are solely those of the authors and do not necessarily represent those of their affiliated organizations, or those of the publisher, the editors and the reviewers. Any product that may be evaluated in this article, or claim that may be made by its manufacturer, is not guaranteed or endorsed by the publisher.
